# DHA-Induced Perturbation of Human Serum Metabolome. Role of the Food Matrix and Co-Administration of Oat β-glucan and Anthocyanins

**DOI:** 10.3390/nu12010086

**Published:** 2019-12-27

**Authors:** Veronica Ghini, Leonardo Tenori, Francesco Capozzi, Claudio Luchinat, Achim Bub, Corinne Malpuech-Brugere, Caroline Orfila, Luigi Ricciardiello, Alessandra Bordoni

**Affiliations:** 1 Center of Magnetic Resonance (CERM), University of Florence, via Luigi Sacconi, 6-50019 Sesto Fiorentino (FI), Italy; ghini@cerm.unifi.it (V.G.); luchinat@cerm.unifi.it (C.L.); 2 Department of Experimental and Clinical Medicine, University of Florence, Largo Brambilla, 3-50134 Florence (FI), Italy; tenori@cerm.unifi.it; 3 Department of Agricultural and Food Sciences, Alma Mater Studiorum University of Bologna, Piazza G. Goidanich, 60, 47521 Cesena (FC), Italy; alessandra.bordoni@unibo.it; 4 Interdepartmental Centre for Industrial Agrofood Research, Alma Mater Studiorum University of Bologna, Via Q. Bucci 336, 47521 Cesena (FC), Italy; 5 Department of Chemistry, University of Florence, via della Lastruccia, 3-50019 Sesto Fiorentino (FI), Italy; 6 GIOTTO Biotech S.r.l., Via Madonna del Piano, 6-50019 Sesto Fiorentino (FI), Italy; 7 Max Rubner-Institute, Federal Research Centre for Nutrition and Food, Haid-und-NeuStrasse 9, DE-76131 Karlsruhe, Germany; achim.bub@mri.bund.de; 8 Université Clermont Auvergne, INRA, UNH, Unité de Nutrition Humaine, CRNH Auvergne, F63000 Clermont-Ferrand, France; corinne.malpuech-brugere@uca.fr; 9 School of Food Science and Nutrition, University of Leeds, Woodhouse Ln, Leeds LS2 9JT, UK; C.Orfila@leeds.ac.uk; 10 Department of Medical and Surgical Sciences, Alma Mater Studiorum University of Bologna, via Massarenti 9, 40138 Bologna (BO), Italy; luigi.ricciardiello@unibo.it

**Keywords:** anthocyanins, bioactive enriched food, docosahexaenoic acid, NMR-based metabolomics, oat beta glucans

## Abstract

Docosahexaenoic acid (DHA) has been reported to have a positive impact on many diet-related disease risks, including metabolic syndrome. Although many DHA-enriched foods have been marketed, the impact of different food matrices on the effect of DHA is unknown. As well, the possibility to enhance DHA effectiveness through the co-administration of other bioactives has seldom been considered. We evaluated DHA effects on the serum metabolome administered to volunteers at risk of metabolic syndrome as an ingredient of three different foods. Foods were enriched with DHA alone or in combination with oat beta-glucan or anthocyanins and were administered to volunteers for 4 weeks. Serum samples collected at the beginning and end of the trial were analysed by NMR-based metabolomics. Multivariate and univariate statistical analyses were used to characterize modifications in the serum metabolome and to evaluate bioactive-bioactive and bioactive-food matrix interactions. DHA administration induces metabolome perturbation that is influenced by the food matrix and the co-presence of other bioactives. In particular, when co-administered with oat beta-glucan, DHA induces a strong rearrangement in the lipoprotein profile of the subjects. The observed modifications are consistent with clinical results and indicate that metabolomics represents a possible strategy to choose the most appropriate food matrices for bioactive enrichment.

## 1. Introduction

Bioactives or nutraceuticals are any substances that are foods, or parts of a food, and provide health benefits, including the prevention and treatment of diseases [1]. Bioactives are usually present in common foods at a low concentration, typically far from the optimally effective dose range. To overcome this, the food and drink industry is developing new products containing high concentrations of selected bioactives, so-called “functional foods”. Functional foods are foods that provide benefits to the body, additional to adequate nutrition, either improving health and well-being or reducing the risk of disease, or both [2]. The majority of them is generated starting from a specific functional ingredient. Thus, the term “functional food” mainly refers to food that is fortified or enriched with bioactive compounds [3].

Bioactives can be considered an integral part of tailored nutrition prescription, therefore, representing a promising approach for both prevention and management of metabolic disorders. Regrettably, meta-analyses evaluating the effects of nutraceuticals seldom discriminate the source of the active compounds, which can be delivered (i) as a dietary supplement, (ii) as a specific dietary treatment (i.e., the increased consumption of food naturally containing the bioactive) or (iii) as a bioactive-enriched food (BEF). In the latter case, the food matrix in which the bioactive is embedded could have a role in the final effect. As an example, other constituents in a food matrix could aid or hinder the bio-accessibility and bioavailability of the bioactives.

In the present study, we investigated by NMR the effects of supplementation with docosahexaenoic acid (C22:6 *n-*3, DHA) on the plasma metabolome of human volunteers at risk of metabolic syndrome (MetS). DHA is a well-known bioactive that has been reported to have a positive impact on many diet-related disease risks, including MetS [4]. DHA was administered as a functional ingredient of three foods. Each food was enriched with DHA alone or in combination with other bioactives that have also been associated with a reduced risk of MetS: Oat beta-glucan (OBG), and anthocyanins (AC) [5,6,7]. The serum metabolome was evaluated by NMR-based metabolomics to investigate the perturbation induced by the dietary treatment, critically evaluating bioactive-food matrix interactions and the extent of possible synergy/antagonism between DHA and the other bioactives.

It has been suggested that metabolomics could play a role in dietary assessment and identification of novel biomarkers of dietary intake [8,9,10,11,12,13,14]. NMR-based metabolomics [15,16,17] are an efficient and highly reproducible platform for the analysis of biofluids. The type and abundance of metabolites detected in a biological sample can be viewed as a global fingerprint that unambiguously describes the current metabolic status of an individual [18,19,20,21]. In the context of the new approach of combining traditional methods with novel metabolomic techniques for well-being and optimal nutrition [22], NMR-metabolomics have shown immense potential for individual monitoring in response to clinical and dietary intervention [23,24,25,26,27].

## 2. Experimental Section

### 2.1. Bioactive Enriched Food 

Three different foods (milkshake, biscuits and pancake), belonging to different food matrices (dairy, bakery and egg-based food), were enriched with DHA, AC, OBG, DHA + AC or DHA + OBG. Each BEF category was produced using the same recipe, with small modifications due to the addition of the active ingredient(s).

DHA, AC and OBG enrichment was obtained by adding OVO-DHA^®^ (Applications Sante des Lipides Sarl, Marseille, France), Eminol^®^ (ABRO BIOTEC SL, Zaragoza, Spain) and SweOat^®^ bran BG28 XF (Swedish Oat Fiber, Väröbacka, Sweden), respectively. Pancake and milkshake were manufactured by production plants coordinated by ADEXGO Kft (Lapostelki utca, Hungary), and biscuits by Desarrollos Panaderos Levantinos SLL (Valencia, Spain). BEF were formulated in order to supply similar daily amounts of bioactives within and among the different food matrices (about 250 mg DHA, 3 g OBG and 50 mg AC). The different matrices appeared not equally compliant to the enrichment with AC due to the sensory and physico-chemical characteristics of the polyphenols, and to their different interactions with the food matrix/food processing. Thus, it was decided to enrich each matrix with the highest possible amount of AC, taking care to maintain good sensory characteristics and consumers’ acceptance of the product. Due to the shelf life and retention of bioactives during storage, BEF were produced in different batches. Bioactive concentrations were measured in at least 3 products in each batch, on the day of production and on the last day of shelf life, and the average amount of bioactives delivered with one portion of BEF is reported in Table 1. DHA and BG content were comparable and not statistically different in BEF from the different matrices, while AC content was different in the order pancake > milkshake > biscuits. Anyway, the amount of AC delivered with BEF within the same matrix (and in the same pilot) was almost the same.

### 2.2. Subjects

The study was conducted by running 3 separate trials, each of them testing one of the 3 BEF categories (milkshake, biscuits or pancake), as reported in Bub et al. [7]. The trials were conducted: (i) Max Rubner-Institut, Karlsruhe, Germany, MRI, (milkshake); (ii) Centre de Recherche en Nutrition Humaine Auvergne, Clermont-Ferrand, France, CRNH (biscuits); (iii) School of Food Science and Nutrition, University of Leeds, Leeds, UK, ULE (pancake).

Enrolled subjects were healthy men and women aged 18 to 80 years presenting 2, 3 or 4 of the criteria for MetS diagnosis [elevated waist circumference ≥102 cm (men) or ≥88 cm (women); fasting triglycerides ≥150 mg/dL; fasting HDL-cholesterol ≤40 mg/dL (men) or ≤50 mg/dL (women); systolic blood pressure ≥130 mmHg and/or diastolic blood pressure ≥85 mmHg or hypotensive treatment; fasting glucose ≥110 mg/dL], with at least one of them being elevated fasting triglycerides (TG) or low high-density lipoprotein cholesterol (HDL-C) [7].

In each trial, volunteers received a BEF belonging to the same matrix (milkshake, biscuits or pancake) for 28 days. Trials were randomized, double-blind, parallel dietary intervention without a placebo. All subjects gave their informed consent for inclusion before they participated in the study. The study was conducted in accordance with the Declaration of Helsinki. Ethical approval for the study protocol was obtained from national authorities: MRI ethical committee approval reference number: F-2014-062 (State Medical Chamber Baden-Wuerttemberg); CRNH Regional Committee ethics reference number: 2014-A01290-47; ULE MEEC-Ethics reference number: MEEC 13-027, amended reference number MEEC 14-017.

In each trial, participants were divided into 5 subgroups, each one receiving a specific enrichment, and were required to consume one portion of the allocated BEF daily, for a total of 4 weeks. Volunteers were on a free diet, apart from the indication to limit the consumption of food naturally containing high amounts of the bioactives (i.e., fish for DHA) to one portion per day and were required to maintain their usual lifestyle during the intervention. At baseline (T0) and after 4 weeks of intervention (T1), fasting blood samples were collected for NMR analysis. Since haemolysis has been reported to interfere with NMR analysis contributing to erroneous results [28], visually haemolysed samples were excluded.

The number of participants having suitable samples for analysis at both T0 and T1 is reported in Table 2.

### 2.3. NMR Sample Preparation

NMR samples were prepared according to standard procedures [29,30]. Frozen serum samples were thawed at room temperature and shaken before use. A total of 350 µL of each sample was added to 350 µL of a phosphate sodium buffer (70 mM Na_2_HPO_4_; 20% (*v/v*) D_2_O; 0.025% (*v/v*) NaN_3_; 0.8% (*w/v*) sodium trimethylsilyl (2,2,3,3-^2^H_4_) propionate (TMSP), pH 7.4). The mixtures were homogenized by vortexing for 30 s, and a total of 600 μL of each mixture was transferred into a 5.00 mm NMR tube (Bruker BioSpin Gmbh, Rheinstetten, Germany) for analysis.

### 2.4. NMR Experiments

^1^H-NMR spectra for all samples were acquired using a Bruker 600 MHz spectrometer (Bruker BioSpin Gmbh, Rheinstetten, Germany) operating at 600.13 MHz proton Larmor frequency and equipped with a 5mm PATXI ^1^H-^13^C-^15^N and ^2^H-decoupling probe including a z-axis gradient coil, an automatic tuning-matching (ATM) and an automatic and refrigerate sample changer (SampleJet, Bruker BioSpin Gmbh, Rheinstetten, Germany). A BTO 2000 thermocouple served for temperature stabilization at the level of approximately 0.1 K at the sample. Before measurement, samples were kept for 5 min inside the NMR probe head for temperature equilibration at 310 K.

For each serum sample, 2 monodimensional ^1^H NMR spectra were acquired with water peak suppression and different pulse sequences that allowed the selective observation of different molecular components: (i) a standard NOESY (Nuclear Overhauser Effect Spectroscopy) [31] 1Dpresat (noesygppr1d.comp; Bruker BioSpin Gmbh, Germany) pulse sequence, using 32 scans, 98,304 data points, a spectral width of 18,028 Hz, an acquisition time of 2.7 s, a relaxation delay of 4 s and a mixing time of 0.01 s. This pulse sequence was designed to obtain a spectrum in which both signals of metabolites and high molecular weight molecules (lipids and lipoproteins) were visible. (ii) a standard CPMG (Carr-Purcell-Meiboom-Gill) [32] (cpmgpr1d.comp; Bruker BioSpin Gmbh, Rheinstetten, Germany) pulse sequence, using 32 scans, 73,728 data points, a spectral width of 12,019 Hz and a relaxation delay of 4 s. This pulse sequence was designed for the selective observation of small molecule components in solutions containing macromolecules.

### 2.5. NMR Spectra Processing and Spectral Analysis

Free induction decays were multiplied by an exponential function equivalent to a 0.3 Hz line-broadening factor before applying a Fourier transform. Transformed spectra were automatically corrected for phase and baseline distortions and calibrated (glucose doublet at δ 5.24 ppm) using TopSpin 3.5 (Bruker BioSpin Gmbh, Rheinstetten, Germany).

Each spectrum, in the region of 10.00–0.2 ppm, was segmented into 0.02 ppm chemical shift buckets, and the corresponding spectral areas were integrated using the AMIX software (Bruker BioSpin Gmbh, Rheinstetten, Germany). Bucketing was a method to decrease the data dimensionality and to compensate for a small shift in the signals, making the analyses more robust and reproducible. The area of each bin was normalized to the total spectral area, calculated with the exclusion of the water region (4.40–5.00 ppm).

### 2.6. Statistical Analysis

Various kinds of multivariate statistical techniques were applied on the obtained buckets using R 3.0.2 in house scripts.

Unsupervised Principal Component Analysis (PCA) was used to obtain a preliminary overview of the data (visualization in a reduced space, clusters detection, screening for outliers). Partial Least Squares (PLS) and Multilevel PLS (M-PLS) was employed to perform supervised data reduction and classification. Canonical analysis (CA) was used in combination with PCA to increase supervised data reduction and classification. The global accuracy for classification was assessed by means of a Monte Carlo validation scheme. Accordingly, each dataset was randomly divided into a training and a test set, including the 90% and 10% of the data, respectively. The training set was used to build the model, whereas the test set was used to validate its discriminant and predictive power; this operation was repeated 500 times. The resultant confusion matrix was reported, and discrimination accuracy, specificity and sensitivity were estimated according to standard definitions.

The metabolites, whose peaks in the spectra were well defined and resolved, were assigned, and their levels analysed. The assignment procedure was made up using an NMR spectra library of pure organic compounds, public databases (e.g., Human Metabolome Database [33]), storing reference NMR spectra of metabolites, spiking NMR experiments and using literature data [34]. The relative concentrations of the various metabolites were calculated by integrating the corresponding signals in the spectra [35].

The pairwise Wilcoxon signed-rank test was used for the determination of the meaningful metabolites; false discovery rate correction was applied using the Benjamini and Hochberg method (FDR): A *p*-value of 0.05 was deemed significant.

## 3. Results

**Figure 1:**
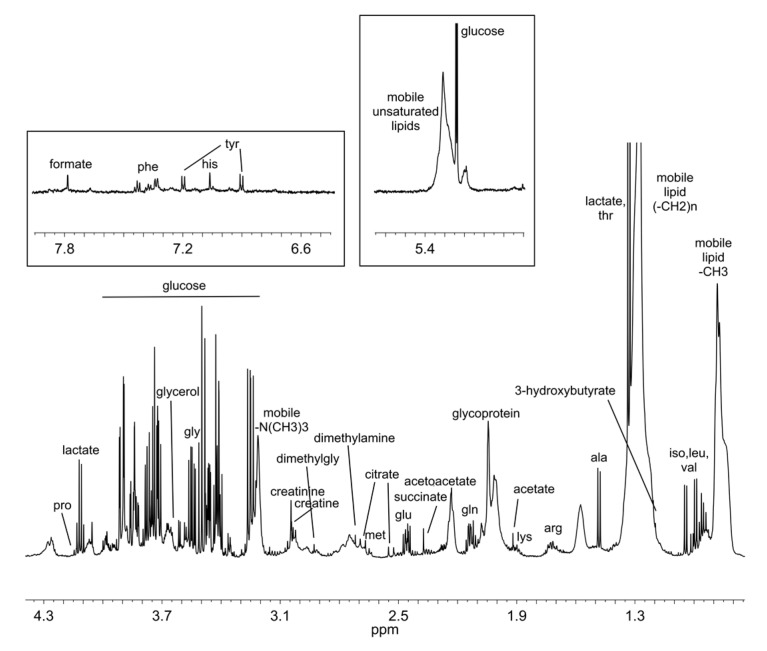
Typical 1H-NMRCPMG spectrum of serum. Most abundant metabolites are labelled.

Untargeted NMR-based metabolomic analysis of serum samples was used to analyse the metabolomics effect of DHA supplementation, alone or in combination with OBG and AC, as an ingredient of the three different BEF. ^1^H CPMG NMR spectra were acquired verifying their high reproducibility. Figure 1 shows a typical CPMG spectrum of a serum sample.

**Figure 2:**
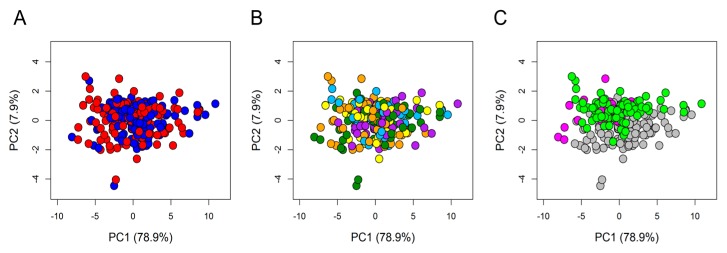
Principal component analysis (PCA) score plot. PC1 and PC2 account for 79.9% and 7.9%, respectively, of the total variance. In the score plot, each dot represents a different serum sample, and each colour represents a different group: (**A**) Blue dots = T0 samples; red dots = T1 samples. (**B**) Dark green dots = anthocyanins (AC); cyan dots = oat beta-glucan (OBG); orange dots = docosahexaenoic acid (DHA); yellow dots = DHA + AC; purple dots = DHA + OBG. (**C**) grey dots = samples from Germany; magenta dots = samples from the UK; light green dots = samples from France.

To assess the quality of the overall spectral dataset, PCA was applied on the 234 × 397 data matrix where 234 was the total number of sera analysed, and 397 was the number of 0.02 chemical shift buckets in which each spectrum was segmented, as described in the Material and Methods. The resulting score plot (Figure 2) evidenced that all samples, although collected in three different trials performed in three different recruiting centres, each one testing a specific food matrix, were well homogenous to each other and no outliers were detected. The uniform distribution of the samples can be easily visualized by colouring the scores according to T0/T1 (Figure 2A), supplements (Figure 2B), and matrix/centres (Figure 2C).

By using supervised PCA—CA analysis to discriminate the five different supplements, separately at T0 and T1, and by considering the prediction accuracy of the models obtained, it turned out that the recruitment protocol selected a very homogenous population, without introducing any meaningful source of variation in the sera metabolome. As expected, considering all the samples collected before the treatment (T0), the confusion matrix was consistent with a random distribution among the different treatments (Figure S1).

After the administration of BEFs (T1), discrimination among groups raised up to 30% (Figure S1), indicating the presence of some modifications of the metabolomic profiles due to the treatments.

A paired (before vs. after treatment) M-PLS analysis was used to consider the possible presence of a strong individual variability in response to the treatments. In this kind of approach, the effects of the treatments can be evaluated within each subject (considering only intra-individual variability), thus eliminating the noise introduced by the inter-individual variability.

The use of M-PLS allowed us to obtain a good classification accuracy (78%) considering the whole dataset. Due to the uneven recruitment in the three trials, it was possible to significantly explore the presence of different food matrix effects only in volunteers allocated to the enriched milkshake. Acting this way, the discrimination accuracy rose up to 89%. Therefore, M-PLS clearly indicated that daily BEF consumption, particularly milkshake, significantly affected the individual metabolome.

To assess whether the observed metabolic changes were merely an unspecific phenomena related to consumption of the BEF matrix, or whether they were specifically related to the increased intake of bioactives, M-PLS was applied to each specific enrichment, considering either all BEF or milkshake only. The resulting classification performances are reported in Table 3.

Considering all food matrices together, supplementation with DHA gave a moderately high discrimination accuracy (74%) between T0 and T1. In contrast, AC and OBG alone did not significantly alter the individual metabolome. Interestingly, co-administration of DHA and AC reduced the metabolomic effects of the fatty acid, while a strong cooperative and synergic effect was detected in volunteers assuming DHA + OBG. In fact, the DHA + OBG enrichment provided the best classification accuracy (86%) that was further improved considering the milkshake group only (94%).

The strong effect of DHA + OBG observed after consumption of the milkshake could not be unequivocally explained by a deeper impact exerted by this specific enrichment-food matrix interaction on the metabolome, because the same analysis could not be significantly repeated on enriched pancake and biscuits due to the very low number of available samples. Nevertheless, even though not reliable, the results obtained for the subjects allocated to enriched biscuits were reported in Table S1 and they were in line with those obtained for the enriched milkshake.

It could be argued that the higher accuracy obtained considering only the milkshake may be simply due to the reduction of noise and variability that were detected when all matrices were included in the analysis.

**Figure 3:**
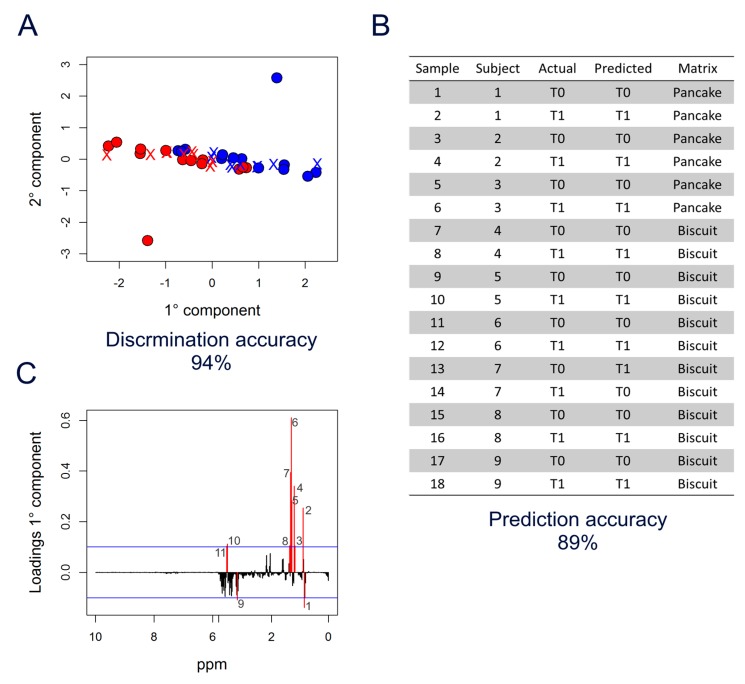
M-PLS analysis of DHA + OBG supplementation. (**A**) M-PLS score plot. Milkshake samples are used as a training set to discriminate T0 (blue dots) vs. T1 (red dots). Biscuit and pancake samples are used as a test set (crosses coloured according to the prediction). (**B**) Table reporting the prediction results. (**C**) M-PLS loading plot of the first component (PC1); the significance threshold (blue lines) was calculated considering “buckets” with a value beyond two standard deviations of their averages; 1: 0.85 ppm—2: 0.89 ppm (CH_3_ VLDL-LDL); 3–4: 1.17–1.19 ppm—5–7: 1.27–1.31 ppm—8: 1.35 ppm ((-CH_2_-)n VLDL-LDL); 9: 3.23 ppm; 10–11: 3.65–3.67 ppm.

Thus, to validate the observed strong effect of DHA + OBG supplementation, the M-PLS discrimination model (T0 vs. T1) built on samples from volunteers consuming the milkshake was used to predict the collection time (T0 or T1) of samples from volunteers receiving the same enrichment (DHA + OBG) embedded in the other food matrices (Figure 3A). Notably, most of the samples (89%) were correctly classified as T0 or T1 (Figure 3B), indicating that the spectral features able to capture the metabolic changes occurring during administration of DHA + OBG were independent of the matrix used as a vehicle. All the six samples from the three subjects allocated to the enriched pancake were perfectly classified (100%), whereas in the case of enriched biscuits, the two samples of one out of the six subjects were mixed up (subject 7 in Figure 3B).

The M-PLS analysis (Figure 3A) was then explored to identify the signals in the spectra that were mainly related to the DHA + OBG effects on the serum metabolome, as depicted in the loading plot of the first component (Figure 3C). The threshold (blue lines) used to select significant signals was calculated considering bins with values beyond two standard deviations of their averages. Interestingly, most of these bins belonged to the broad signals in the spectra arising from methyl (-CH_3_) and methylene (-CH_2_-) groups of serum lipoproteins, centred at 0.86 and 1.33 ppm, respectively (Figure 1).

Accordingly, M-PLS analysis applied on the small region of ^1^H-NOESY spectra containing the broad signal attributable to the resonances of lipoprotein methyl groups (0.92–0.71 ppm) resulted in a discrimination accuracy of 78% (DHA + OBG group, T0 vs. T1) (Figure S2). This result confirms that DHA + OBG administration significantly changes the lipoprotein profiles.

The broad signals of methyl and methylene groups arise from the sum of partial overlapping peaks of the different fractions (HDL, LDL, IDL, VLDL and chylomicrons). Due to the different density of the lipoprotein fractions, their chemical shift differences are quite small but significantly different. Thus, in each of the two broad signals, there is a direct correlation between chemical shift and lipoprotein particle size and density, with the smaller and denser particles (HDL) contributing to a farther upfield part of the signals. In contrast, chylomicrons and VLDL, the bigger and less dense fractions, contribute to the farther downfield part of the signals [36,37,38].

**Figure 4:**
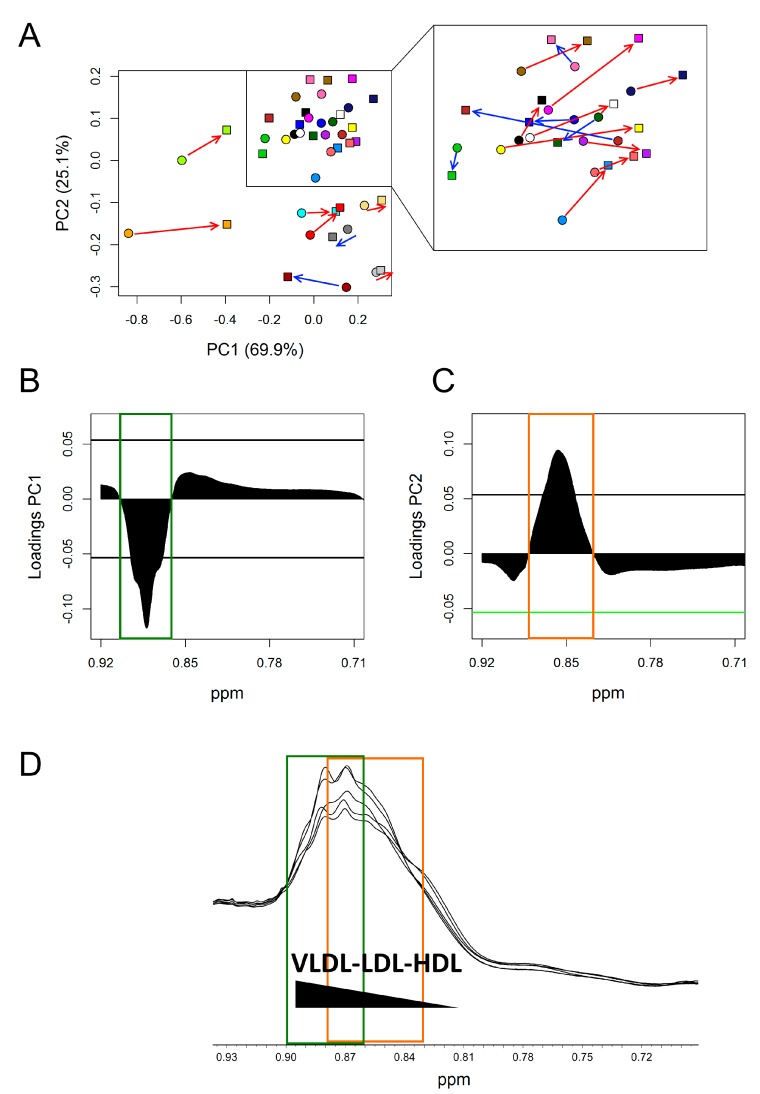
PCA analysis of lipoprotein methyl group signal (CH_3_) (0.92–0.71 ppm) of DHA + OBG group. (**A**) Score plots; each colour represents a different subject at TO (dots) and T1 (squares). Red arrows: Subjects (15 out of 22) that, going from T0 to T1, move towards more positive value along both PC1 and PC2; blue arrows: Subject that moves towards more negative value along PC1. (**B**) PC1 loading plot. (**C**) PC2 loading plot. In both loading plots, the threshold (black horizontal lines) used to select significant parts of the methyl signal was calculated considering the spectral area with values beyond two standard deviations of their averages. (**D**) ^1^H-NOESY spectral area containing lipoprotein methyl signal. Green square: Significant spectral area in PC1 loading plot; orange square significant spectral area in PC2 loading plot.

To investigate the observed changes more in depth, an unsupervised PCA model was built on the small spectral region selected above (0.92–0.71 ppm) (Figure 4). As an effect of DHA + OBG treatment, most of the subjects (15 out of 22) moved towards more positive values along with both principal component PC1 and PC2, going from T0 to T1 (Figure 4A). In all subjects, the variation along PC1 was much stronger than the variation along PC2. Principal component loadings were then explored to identify in detail which portions of the broad methyl signal were mainly responsible for the changes observed in the score plot for the subjects shifting towards more positive values. The PC1 loading plot (Figure 4B,D) clearly showed a significant decrement of the spectral area between 0.905–0.862 ppm, centred at 0.884 ppm, attributable to chylomicrons and VLDL fraction resonances. The PC2 loading plot, instead, showed an increment of the spectral area between 0.882 and 0.829 ppm, centred at 0.858 ppm, attributable to LDL resonances (Figure 4C,D).

The significant decrement (*p* = 0.004) of the signal attributable to triglycerides (TG) resonance, in T1 ^1^H-NOESY spectra of DHA + OBG group compared to baseline (T0) confirmed the reduction of chylomicrons and VLDL. M-PLS analysis applied only on the small part of DHA + OBG spectra containing the broad TG signal (4.34–4.22 ppm), which resulted in a very high (80%) discrimination accuracy between T0 and T1 (Figure S3).

^1^H-NOESY spectra were also analysed using the Bruker IVdr Lipoprotein subclass analysis (B.I. -LISA) platform [39]. The results for all BEF samples together are reported in Table 4 and Table S2 and are in agreement with previously reported data. Considering single bioactives, no modification in the lipoprotein profile was observed in volunteers receiving AC or OBG. On the contrary, DHA administration was characterised by a significant increase of total ApoB100 and LDL particle number; the observed LDL increase was mainly due to the increase of small, dense LDL (LDL 4+5).

Volunteers receiving DHA + OBG showed a different and strong rearrangement in the lipoprotein profile after the dietary intervention. This group was characterized by a significant decrement of VLDL particle number and TG. The decrement of TG was associated with TG decrease in VLDL, IDL, HDL and LDL1, without any modification in LDL4 and 5 subparticles.

No modification in the lipoprotein profile was also observed in volunteers receiving DHA + AC.

Since DHA + OBG administration was by far the treatment inducing the most relevant modifications, changes in other metabolite levels were also investigated. The signals of 29 metabolites were unambiguously assigned and integrated with the ^1^H-NMR spectra of the sera. All the assigned metabolites are listed in Table S3, with the respective median concentration at T0 and T1. Despite significant changes in the concentration of some metabolites, the modification in the sera metabolomic profile observed after the consumption of food enriched with DHA + OBG remains mainly attributable to lipoprotein rearrangement.

## 4. Discussion

In this study, the metabolic effects of DHA supplementation, alone or in combination with OBG and AC, as an ingredient of the three different BEF, were characterized by using NMR-based metabolomics. We clearly evidenced that AC or OBG alone did not cause significant changes in the serum metabolome, which was significantly modified by DHA. Interestingly, co-administration of AC or OBG influenced DHA-induced modifications (Table 3). Using the same analytical approach, we have recently evidenced similar effects in the lipidome and metabolome of cultured human hepatocytes [40].

Dietary intervention with DHA was characterised by the increase of total ApoB100 and LDL particle number, mainly the smaller and denser sub-particles LDL 4 and LDL 5 (Table 4). Omega-3 fatty acid is highly effective and generally well-tolerated TG lowering agents. However, they are reported to increase LDL [41]. A modest increase of LDL level after DHA administration is also reported in clinical trials, but since the clinical relevance of such findings is uncertain [42] and given the advocated benefits, it is relevant to determine whether the increase in LDL is offset by the improvement of LDL particle size. Although DHA supplementation induced a shift toward smaller LDL particles having greater atherogenicity [43], it also decreased the apolipoprotein B100 (ApoB100)/apolipoprotein A1 (ApoA1) ratio. Apo B100 is present in atherogenic lipoproteins VDL, IDL and LDL, while ApoA1 is a major constituent of HDL. Thus, the ApoB100/ApoA1 ratio represents the balance between atherogenic and anti-atherogenic lipoproteins. Since several studies have reported that this ratio is associated with MetS risk independently of conventional risk factors [44,45], we argue that the 4-week consumption of DHA-enriched BEF decreased the risk of MetS.

Interestingly, AC co-administration completely annulled the DHA effect (Table 3 and Table 4). On the contrary, when co-administered with oat beta-glucan, DHA induces a different and very strong rearrangement of the lipoprotein profile (Figure 3, Table 3 and Table 4). These metabolomic perturbations are consistent with the clinical effects observed in the trial [7] and with the already reported effect of DHA, which is supposed to decrease TG concentrations by reducing TG synthesis, incorporation into VLDL, and secretion, and by enhancing TG clearance from VLDL particles [46]. To the authors’ knowledge, this is the first report indicating a synergism of DHA and OBG. The combined effect of these two bioactives has been only tested in the CONFIDENCE study [47], which results are not available yet.

The uneven recruitment in the three trials did not permit to explore the presence of different food matrix effects fully. Notwithstanding, considering only samples from the milkshake trial a higher M-PLS discrimination accuracy was evidenced (Table 3), letting us speculate that the food matrix in which DHA is embedded also has a role in the final effect. This could be related to the modification of DHA bioavailability. Supplemented DHA must be absorbed and distributed into tissues to exert its effect, and the food matrix, including the co-enrichment with AC or OBG, could have a role. The differential modification of DHA serum level after the consumption of the different BEF [7] confirms this hypothesis and highlights that the concentration of DHA in food cannot be considered as a univocal index of functionality since many other factors contribute to the final effect. Nevertheless, the M-PLS discrimination model (T0 vs. T1) built on samples from volunteers consuming the DHA + OBG milkshake allowed us the correct prediction of the collection time (T0 or T1) of test samples from volunteers receiving the same enrichment embedded in the other food matrices (Figure 3), indicating the coherence of the detected changes, as the robustness of the model built on the DHA + OBG milkshake consumption overcomes the “noise effect” generated by the other matrixes.

## 5. Concluding Remarks

Nutritional factors contribute to the movements of an individual in the metabolic space [19], and with only considering this tuning in a multidimensional space, we will have the possibility of understanding the complex changes due to diet in a foodomic vision [48]. Results herein reported evidence that NMR-based metabolomics can characterise modifications in the metabolome consequent to the administration of bioactives as ingredients of BEF. Using this approach, we demonstrated that the metabolome perturbation induced by DHA administration is attributable to a rearrangement in the lipoprotein profile and it is influenced by the food matrix and the co-presence of other bioactives. In particular, the co-administration of DHA + AC reduced the metabolomic effects of the fatty acid alone, while a strong cooperative and synergic effect was monitored in the case of co-administration of DHA + OBG.

Modifications observed in the metabolome were consistent with clinical results of the trial that evidenced a significant decrease in serum TG in volunteers receiving DHA + OBG-enriched milkshake [7]. Metabolomics allowed relating TG decrease to the modification in lipoprotein profile, which could better predict modification in disease risk. Notably, regardless of the source (fish, fish oil, algae, supplements, etc.) the median dose required to obtain a clinical effect is reported to be above 1.5 g DHA/day [49,50]. In the present study, the daily dose of DHA was lower than 0.3 g; notwithstanding its administration as an ingredient of BEF, particularly when in combination with OBG, modified the serum metabolome in human volunteers at risk of MetS.

The absence of a placebo group receiving not enriched food should not be considered as a limitation of this study since all BEF within the same matrix had the same composition apart from the addition of the different bioactives. BEF containing AC or OBG alone did not significantly alter the individual metabolome and can be considered as controls, thus excluding that the observed effects were simply due to the consumption of the foods even in the absence of supplementation.

## Figures and Tables

**Table 1:** The average amount of bioactives delivered with one portion of bioactive-enriched foods (BEF).

	DHA (mg)	AC (mg)	OBG (g)
Biscuits DHA	292	0	0
Biscuits DHA + AC	302	19	0
Biscuits DHA + OBG	329	0	2.9
Biscuits AC	0	17	0
Biscuits OBG	0	0	3.6
Pancake DHA	225	0	0
Pancake DHA + AC	208	57	0
Pancake DHA + OBG	215	0	4.3
Pancake AC	0	58	0
Pancake OBG	0	0	3.7
Milkshake DHA	261	0	0
Milkshake DHA + AC	228	12	0
Milkshake DHA + OBG	226	0	3.8
Milkshake AC	0	10	0
Milkshake OBG	0	0	4.2

**Table 2:** Number of study participants according to treatment.

Matrix	Subjects (TOT)	DHA	AC	OBG	DHA + AC	DHA + OBG
Milkshake	66	14	15	12	12	13
Biscuits	37	7	7	9	8	6
Pancake	14	5	3	1	2	3
Total	117	26	25	22	22	22

**Table 3:** Multilevel partial least squares (M-PLS) discrimination accuracy values.

	All BEF	Enriched Milkshake
DHA	74% **	71% *
AC	63% *	54%
OBG	55%	55%
DHA + AC	53%	56%
DHA + OBG	86% **	94% **

Accuracies significantly above the chance level of 50% (binomial test) are marked with: * 0.001 < *p*-value <0.05; ** *p*-value <0.001.

**Table 4:** Bruker IVdr Lipoprotein subclass analysis.

	DHA		DHA + OBG	
	T0	T1	T0	T1
TG	156.48	138.83	193.56	153.63 *
Chol	226.25	240.43 *	252.82	250.90
LDL-Chol	122.91	129.37	136.43	147.09 *
Apo B100	100.41	108.63 *	114.74	113.23
Apo A2	32.71	32.84	35.42	34.98 *
Calculated Figures				
Apo B100/ Apo A1	1.42	1.26 *	1.27	1.29
Total Apo B100 Particle Number	1825.71	1975.17 *	2086.31	2058.91
VLDL Particle Number	192.87	175.21	236.63	208.71 *
LDL Particle Number	1496.1	1589.47 *	1703.64	1726.34
Lipoprotein Main Fractions				
TG-VLDL	105.3	92.49	130.67	112.03 *
TG-IDL	17.61	14.07	23.67	16.52 *
TG-LDL	22.05	25.26 *	24.55	22.80
TG-HDL	10.69	10.97	11.69	10.10 *
Chol-VLDL	27.59	22.44	34.54	28.24 *
Chol-IDL	15.97	16.93	19.76	15.57 *
Chol-LDL	122.91	129.37	136.43	147.09 *
Free Chol-VLDL	12.65	11.27	15.23	12.54 *
Free Chol-IDL	4.48	4.95	5.48	4.47 *
Phospholipids-VLDL	28.25	24.50	33.71	28.06 *
Phospholipids-IDL	8.67	8.925	12.03	10.23 *
Phospholipids-LDL	69.93	72.97 *	75.48	81.04
Apo A2-HDL	33.53	33.99	36.26	35.63 *
Apo B-VLDL	10.61	9.63	13.01	11.48 *
Apo B-LDL	82.28	87.42 *	93.7	94.94
VLDL Subfractions				
TG-VLDL 1	49.54	42.19	62.56	55.9 *
TG-VLDL 2	19.73	13.31	23.62	19.14 *
TG-VLDL 3	15.46	12.54	18.71	16.97 *
TG-VLDL 4	10.82	9.63	13.48	12.31 *
TG-VLDL 5	3.50	3.4	3.59	3.31 *
Chol-VLDL 1	9.93	8.89	11.59	9.13 *
Chol-VLDL 2	4.68	3.73	5.67	4.87 *
Free Chol-VLDL 1	3.51	3.32	4.11	3.98 *
Free Chol-VLDL 2	1.93	1.67	2.31	1.93 *
Free Chol-VLDL 3	2.07	1.69	2.55	2.42 *
Phospholipids-VLDL 1	8.43	6.92	10.17	9.39 *
Phospholipids-VLDL 2	4.81	3.71	5.86	4.905 *
Phospholipids-VLDL 3	4.83	4.09	5.91	5.74 *
LDL Subfractions				
TG-LDL 1	6.57	6.42	6.84	6.1 *
TG-LDL 4	2.76	2.88 *	3.00	3.03
TG-LDL 5	3.13	3.79 *	3.94	4.01
Apo A2-HDL 2	2.98	3.19	3.74	3.57 *
Apo A2-HDL 3	6.49	6.68	7.43	6.95 *
HDL Subfractions				
Free Chol-HDL 2	1.73	1.68	1.8	1.69 *
Free Chol-HDL 3	2.33	2.47	2.70	2.29 *
Free Chol-HDL 4	4.57	4.475	4.97	4.41 *
Phospholipids-HDL 3	15.59	15.15	15.94	16.25 *
Apo A1-HDL 2	16.43	17.87 *	18.35	17.33
Apo A1-HDL 3	25.77	26.76	26.60	26.46 *
TG-HDL 2	1.69	1.79	1.67	1.415 *
TG-HDL 3	2.33	2.5	2.7	2.11 *
TG-HDL 4	4.24	4.105	4.61	4.01 *

Only parameters which resulted statistically significant in the comparison T0 vs. T1, in DHA and DHA + OBG groups, are reported. * for *p* < 0.05.

## Supplementary Materials

The following are available online at https://www.mdpi.com/2072-6643/12/1/86/s1. Figure S1: 5-Group discrimination. Score plot of PCA-CA analysis at T0 (a) and T1 (b). The values of the discrimination accuracy and the confusion matrices are also reported. In the score plot, each dot represents a different serum sample, and each colour represents a different group: dark green dots= AC; cyan dots= OBG; orange dots= DHA; yellow dots= DHA+AC; purple dots= DHA+OBG; Figure S2: Score plot of M-PLS analysis (T0 vs. T1) of the spectral region (0.92–0.71 ppm) containing the signal of lipoprotein methyl group (-CH3) resonances. The value of the discrimination accuracy is also reported. In the score plots, each dot represents a different serum sample of DHA+OBG group, and each colour represents a time point: blue dots= T0 samples; red dots= T1 samples; Figure S3. Score plot of M-PLS analysis (T0 vs. T1) of the spectral region (4.34–4.22 ppm) containing the signal of triglyceride resonances. The value of the discrimination accuracy is also reported. In the score plots, each dot represents a different serum sample of DHA+OBG group, and each colour represents a time point: blue dots= T0 samples; red dots= T1 samples; Table S1. M-PLS discrimination accuracy values; Table S2: Bruker IVdr Lipoprotein subclass analysis. Each significant comparison (T0 vs T1) is indicated with * for *p* < 0.05 and ** *p* < 0.01; Table S3: List of metabolites whose signals were assigned and integrated in the NMR spectra. DHA+OBG discrimination between T0 and T1 in all samples and only in milkshake; each significant comparison (T0 vs T1) is indicated with * for *p* < 0.05 and ** *p* < 0.01.
