# Supplementary material for: DHA-Induced Perturbation of Human Serum Metabolome. Role of the Food Matrix and Co-Administration of Oat β-glucan and Anthocyanins
Source: Nutrients. 2019 Dec 27;12(1):86. doi: 10.3390/nu12010086 (PMC7019822; doi:10.3390/nu12010086)
Supplement: Supplementary file 1 [file nutrients-12-00086-s001.pdf]

DHA-induced perturbation of human serum metabolome. Role of the food matrix and co-administration of oat β-glucan and anthocyanins

Supplementary Materials

Veronica Ghini[1], Leonardo Tenori[2], Francesco Capozzi[3]*, Claudio Luchinat[1,4,5], Achim Bub[6], Corinne Malpuech-Brugere[7], Caroline Orfila[8], Luigi Ricciardiello[9], Alessandra Bordoni[3]

[1] Center of Magnetic Resonance (CERM), University of Florence, via Luigi Sacconi, 6-50019 Sesto Fiorentino (FI), Italy.

[2] Department of Experimental and Clinical Medicine, University of Florence, Largo Brambilla, 3-50134 Florence (FI), Italy.

[3] Department of Agro-Food Science and Technology, University of Bologna, Piazza Goidanich, 60, Cesena (FC), Italy;

[4] Department of Chemistry, University of Florence, via della Lastruccia, 3-50019 Sesto Fiorentino (FI), Italy;

[5] GIOTTO Biotech S.r.l., Via Madonna del Piano, 6-50019 Sesto Fiorentino (FI), Italy;

[6] Max Rubner-Institute, Federal Research Centre for Nutrition and Food, Haid-und-NeuStrasse 9, DE-76131, Karlsruhe, Germany.

[7] Université Clermont Auvergne, INRA, UNH, Unité de Nutrition Humaine, CRNH Auvergne, F63000 Clermont-Ferrand, France.

[8] School of Food Science and Nutrition, University of Leeds, Woodhouse Ln, Leeds LS2 9JT, UK.

[9] Department of Medical and Surgical Sciences, University of Bologna, via Massarenti 9, Bologna (BO), Italy.

* Correspondence: francesco.capozzi@unibo.it; Tel.: +39 0547 338106.

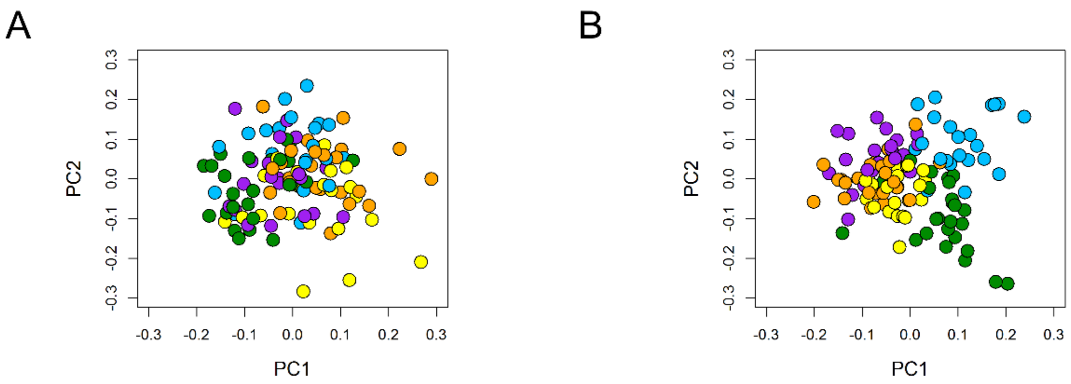

	AC	BG	DHA	DHA+AC	DHA+BG
**AC**	**18.6**	23.3	8.4	36.1	13.5
**BG**	19.3	**14.5**	15.3	13.5	37.3
**DHA**	6.3	24.7	**26.5**	15.1	27.4
**DHA+AC**	27.9	13.4	22	**17**	19.7
**DHA+BG**	22.1	19.1	20.3	17.2	**21.3**

Discrimination accuracy
19.7%

	AC	BG	DHA	DHA+AC	DHA+BG
**AC**	**26.1**	19.9	14.9	12.5	26.6
**BG**	23.7	**15.8**	24.9	6.6	29
**DHA**	10.3	12	**50.5**	14.9	12.4
**DHA+AC**	14.3	18.7	24.5	**34.4**	8.2
**DHA+BG**	22.1	33.3	8.9	15.9	**19.8**

Discrimination accuracy
30.0%

Figure S1. 5-Group discrimination. Score plot of PCA-CA analysis at T0 (a) and T1 (b). The values of the discrimination accuracy and the confusion matrices are also reported. In the score plot, each dot represents a different serum sample, and each colour represents a different group: dark green dots= AC; cyan dots= OBG; orange dots= DHA; yellow dots= DHA+AC; purple dots= DHA+OBG.

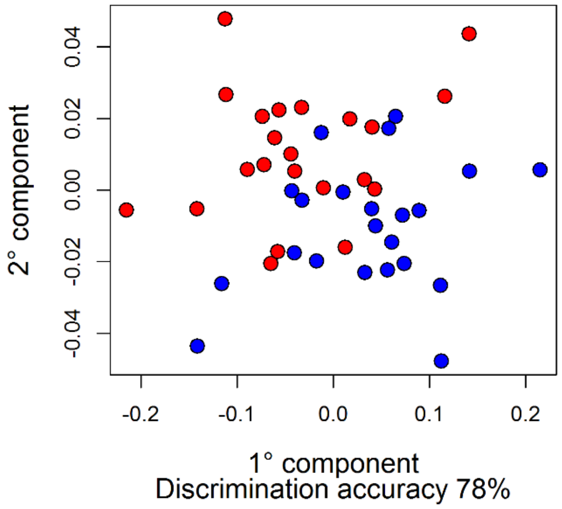

Figure S2. Score plot of M-PLS analysis (T0 vs. T1) of the spectral region (0.92 - 0.71 ppm) containing the signal of lipoprotein methyl group ($-CH_3$) resonances. The value of the discrimination accuracy is also reported. In the score plots, each dot represents a different serum sample of DHA+OBG group, and each colour represents a time point: blue dots= T0 samples; red dots= T1 samples.

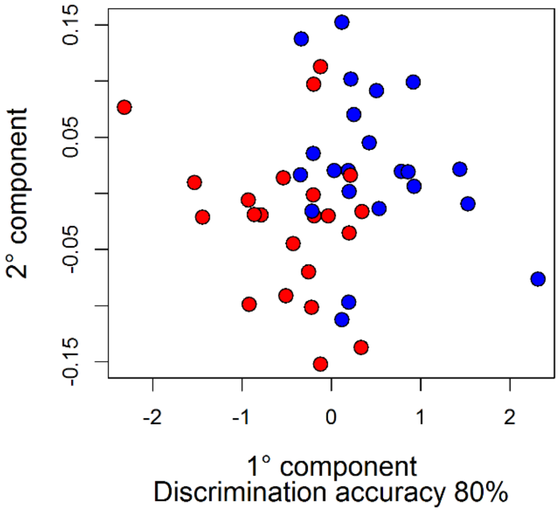

Figure S3. Score plot of M-PLS analysis (T0 vs. T1) of the spectral region (4.34 – 4.22 ppm) containing the signal of triglyceride resonances. The value of the discrimination accuracy is also reported. In the score plots, each dot represents a different serum sample of DHA+OBG group, and each colour represents a time point: blue dots= T0 samples; red dots= T1 samples.

Table S1. M-PLS discrimination accuracy values.

	**Enriched Biscuits**
**DHA**	85%
AC	48%
OBG	66%
DHA+AC	50%
**DHA+OBG**	100%

Table S2. Bruker IVdr Lipoprotein subclass analysis. Each significant comparison (T0 vs T1) is indicated with * for p < 0.05 and ** p < 0.01.

	DHA		AC		OBG		DHA + AC		DHA + OBG	
	T0	T1	T0	T1	T0	T1	T0	T1	T0	T1
**TG**	156.48	138.83	168.67	176.2	166.39	166.96	160.29	162.52	193.56	153.63*
**Chol**	226.25	240.43*	228.49	218.60	239.18	241.74	237.95	230.67	252.82	250.90
**LDL-Chol**	122.91	129.37	123.37	114.94	131.35	129.25	133.44	134.43	136.43	147.09*
**HDL-Chol**	52.04	53.515	48.89	49.17	49.77	51.13	49.47	47.85	50.41	51.50
**Apo A1**	147.1	148.06	147.03	143.32	147.58	149.97	140.9	137.12	146.04	142.07
**Apo A2**	32.71	32.84	32.66	33.52	35.77	34.15	32.36	30.83	35.42	34.98*
**Apo B100**	100.41	108.63*	100.49	104.33	105.43	115.31	111.06	106.76	114.74	113.23
Calculated Figures										
**LDL-Chol/HDL-Chol**	2.40	2.52	2.35	2.325	2.66	2.58	2.64	2.6	2.745	2.53
**Apo B100/ Apo A1**	1.42	1.26*	1.43	1.33	1.43	1.50	1.26	1.30	1.27	1.29
**Total Apo B100 Particle Number**	1825.71	1975.17*	1827.19	1896.97	1916.98	2096.64	2019.42	1941.34	2086.31	2058.91
**VLDL Particle Number**	192.87	175.21	226.40	229.415	203.68	210.67	210.08	198.28	236.63	208.71*
**IDL Particle Number**	114.72	125.52	111.30	110.66	112.87	121.54	133.99	120.06	140.01	113.54
**LDL Particle Number**	1496.1	1589.47*	1498.29	1485.65	1574.71	1641.95	1660.61	1576.62	1703.64	1726.34
**LDL 1 Particle Number**	232.84	261.34	197.92	192.17	229.24	224.15	222.97	211.18	236.15	234.76
**LDL 2 Particle Number**	135.96	146.20	127.89	107.465	144.12	144.61	156.2	149.43	128.89	136.96
**LDL 3 Particle Number**	168.93	175.43	150.52	112.33	172.15	154.01	186.58	188.54	162.15	168.93
**LDL 4 Particle Number**	236.97	250.86	202.88	177.61	233.12	219.68	263.76	259.80	218.57	257.18
**LDL 5 Particle Number**	282.27	340.76	295.64	335.28	294.8	362.03	334.17	327.82	324.57	356.65
**LDL 6 Particle Number**	417.67	476.1	478.34	531.35	458.74	513.02	486.81	441.21	538.21	505.63
Lipoprotein Main Fractions										
**TG-VLDL**	105.3	92.49	113.92	126.08	121.77	115.56	104.23	108.85	130.67	112.03*
**TG-IDL**	17.61	14.07	19.86	19.62	19.45	19.15	17.51	16.67	23.67	16.52*
**TG-LDL**	22.05	25.26*	20.805	22.585	23.32	23.16	24.9	23.22	24.55	22.80
**TG-HDL**	10.69	10.97	10.85	11.52	10.46	9.71	10.84	10.21	11.69	10.10*
**Chol-VLDL**	27.59	22.44	33.35	34.38	29.47	28.26	29.04	27.21	34.54	28.24*
**Chol-IDL**	15.97	16.93	16.4	16.78	16.02	17.52	18.51	16.27	19.76	15.57*
**Chol-LDL**	122.91	129.37	123.37	114.94	131.35	129.25	133.44	134.43	136.43	147.09*
**Chol-HDL**	52.04	53.51	48.895	49.175	49.77	51.13	49.47	47.85	50.41	51.50
**Free Chol-VLDL**	12.65	11.27	14.71	14.13	13.13	13.59	12.56	12.93	15.23	12.54*
**Free Chol-IDL**	4.48	4.95	4.615	4.71	4.59	5	5.33	4.595	5.48	4.47*
**Free Chol-LDL**	36.23	36.81	34.575	32.25	38.09	34.93	37.02	37.78	39.24	41.04
**Free Chol-HDL**	12.46	13.29	11.69	10.95	12.15	11.33	11.68	11.2	12.66	11.89
**Phospholipids-VLDL**	28.25	24.50	31.355	32.84	30.71	31.17	29.44	29.69	33.71	28.06*
**Phospholipids-IDL**	8.67	8.925	9.945	10.335	10.36	9.51	10.75	9.01	12.03	10.23*
**Phospholipids-LDL**	69.93	72.97*	67.985	64.355	75.3	72.86	75.47	73.96	75.48	81.04
**Phospholipids-HDL**	71.10	73.07	69.76	68.11	69.69	74.35	68.24	66.52	70.88	70.82
**Apo A1-HDL**	142.37	144.01	142.035	141.075	145.98	145.79	135.37	130.66	137.75	138.77
**Apo A2-HDL**	33.53	33.99	33.575	34.265	36.38	35.12	33.05	31.83	36.26	35.63*

**Apo B-VLDL**	10.61	9.63	12.45	12.62	11.2	11.59	11.55	10.90	13.01	11.48*
**Apo B-IDL**	6.31	6.90	6.125	6.085	6.21	6.68	7.37	6.60	7.7	6.245
**Apo B-LDL**	82.28	87.42*	82.4	81.71	86.61	90.3	91.33	86.71	93.7	94.94
VLDL Subfractions										
**TG-VLDL 1**	49.54	42.19	53.345	60.545	54.47	50.86	46.315	46.12	62.56	55.9*
**TG-VLDL 2**	19.73	13.31	19.99	22.74	19.33	19.7	19.805	18.32	23.62	19.14*
**TG-VLDL 3**	15.46	12.54	17.975	18	17.3	16.04	16.29	14.33	18.71	16.97*
**TG-VLDL 4**	10.82	9.63	12.345	13.975	11.74	11.53	12.81	11.53	13.48	12.31*
**TG-VLDL 5**	3.50	3.4	3.425	3.13	3.35	3.53	3.17	3.32	3.59	3.31*
**Chol-VLDL 1**	9.93	8.89	10.37	9.765	10.78	10.22	9.37	9.86	11.59	9.13*
**Chol-VLDL 2**	4.68	3.73	5.115	5.425	5.06	4.76	4.65	4.34	5.67	4.87*
**Chol-VLDL 3**	4.45	4.04	5.215	6	5.15	4.62	5.52	4.67	6.10	5.62
**Chol-VLDL 4**	6.21	6.9	7.57	7.26	6.42	6.68	7.75	6.52	8.27	6.48
**Chol-VLDL 5**	1.76	1.59	1.64	1.69	1.46	1.88	1.61	1.64	1.68	1.62
**Free Chol-VLDL 1**	3.51	3.32	3.73	3.92	3.54	3.99	3.71	3.86	4.11	3.98*
**Free Chol-VLDL 2**	1.93	1.67	2.11	2.225	2.15	1.9	1.95	1.74	2.31	1.93*
**Free Chol-VLDL 3**	2.07	1.69	2.485	2.64	2.39	2.17	2.32	1.91	2.55	2.42*
**Free Chol-VLDL 4**	2.51	2.87	2.735	2.72	2.39	2.45	2.98	2.5	3.09	2.37
**Free Chol-VLDL 5**	0.83	0.75	0.84	0.95	0.85	0.94	0.66	0.73	0.88	0.63
**Phospholipids-VLDL 1**	8.43	6.92	8.765	9.88	9.1	8.7	8.11	8.44	10.17	9.39*
**Phospholipids-VLDL 2**	4.81	3.71	5.1	5.515	5.09	4.88	5.08	4.65	5.86	4.905*
**Phospholipids-VLDL 3**	4.83	4.09	5.86	6.195	5.6	5.5	5.60	5.19	5.91	5.74*
**Phospholipids-VLDL 4**	5.58	5.57	6.305	6.89	5.76	5.72	6.75	5.76	6.8	6.04
**Phospholipids-VLDL 5**	2.30	2.06	2.16	2.14	2.04	2.23	2.13	2.05	2.305	1.94
LDL Subfractions										
**TG-LDL 1**	6.57	6.42	5.85	5.91	5.55	6.39	6.63	6.01	6.84	6.1*
**TG-LDL 2**	2.16	2.47	1.96	2.03	2.16	2	2.72	2.51	2.3	2.14
**TG-LDL 3**	2.54	2.735	2.55	2.53	2.92	2.85	3.02	2.96	2.98	2.95
**TG-LDL 4**	2.76	2.88*	2.625	2.665	2.93	2.81	3.08	2.99	3.00	3.03
**TG-LDL 5**	3.13	3.79*	3.69	4.105	3.69	4.21	4.23	3.97	3.94	4.01
**TG-LDL 6**	4.16	4.87	4.865	5.605	4.95	4.84	5.17	5.04	5.39	5.42
**Chol-LDL 1**	24.47	27.52	20.345	19.71	23.52	22.86	24.48	21.78	24.05	24.05
**Chol-LDL 2**	12.69	14.44	12.04	9.26	13.49	13.12	14.89	14.15	12.38	12.83
**Chol-LDL 3**	15.71	16.03	13.43	9.46	15.14	14.22	17.16	16.4	13.43	14.5
**Chol-LDL 4**	20.24	21.23	15.67	14.68	20.13	19.35	22.45	22.54	18.11	21.15
**Chol-LDL 5**	22.34	25.72	23.79	25.87	23.5	27.32	26.53	25.70	24.98	27.01
**Chol-LDL 6**	27.9	32.68	32.14	34.89	32.19	34.64	32.36	29.87	35.63	34.76
**Free Chol-LDL 1**	7.87	8.60	6.64	6.23	7.44	7.04	7.60	6.91	7.845	7.36
**Free Chol-LDL 2**	4.34	4.68	4.22	3.35	4.56	3.96	4.53	4.26	4.18	4.61
**Free Chol-LDL 3**	5.19	5.18	4.49	3.63	5.11	4.52	5.36	4.95	5.35	5.16
**Free Chol-LDL 4**	5.88	6.05	4.53	4.24	5.93	5.46	6.24	6.14	5.50	6.21
**Free Chol-LDL 5**	6.08	6.66	6.22	6.64	5.99	7.4	6.99	6.64	6.63	7.06
**Free Chol-LDL 6**	6.51	7.32	8.16	8.03	7.86	8.28	7.99	7.18	8.83	8.42
**Phospholipids-LDL 1**	13.54	15.39	11.63	11.19	13.36	13.12	13.52	12.46	13.85	13.48
**Phospholipids-LDL 2**	7.30	8.05	7.05	5.34	7.59	7.49	8.27	8.16	7.08	7.57

**Phospholipids-LDL 3**	9.01	8.97	7.88	6.03	8.6	8.09	9.43	9.38	7.78	8.2
**Phospholipids-LDL 4**	11.45	12.20	9.09	8.51	11.03	10.64	12.2	12.35	10.08	11.69
**Phospholipids-LDL 5**	11.90	13.72	12.84	13.78	12.58	14.88	14.33	13.66	13.23	14.41
**Phospholipids-LDL 6**	15.04	16.93	16.89	18.39	17.63	18.59	17.31	16.1	19.04	18.39
**Apo B-LDL 1**	12.80	14.37	10.88	10.57	12.61	12.33	12.26	11.61	12.98	12.91
**Apo B-LDL 2**	7.48	8.04	7.03	5.91	7.93	7.95	8.59	8.21	7.09	7.53
**Apo B-LDL 3**	9.29	9.65	8.28	6.17	9.47	8.47	10.26	10.37	8.91	9.29
**Apo B-LDL 4**	13.03	13.8	11.15	9.77	12.82	12.08	14.51	14.29	12.02	14.14
**Apo B-LDL 5**	15.52	18.74	16.26	18.44	16.21	19.91	18.38	18.03	17.85	19.61
**Apo B-LDL 6**	22.97	26.18	26.31	29.22	25.23	28.21	26.77	24.26	29.6	27.81
HDL Subfractions										
**TG-HDL 1**	2.58	2.93	2.86	2.62	2.44	1.95	2.575	2.61	2.46	2.695
**TG-HDL 2**	1.69	1.79	1.67	1.85	1.49	1.48	1.765	1.66	1.67	1.415*
**TG-HDL 3**	2.33	2.5	2.455	2.6	2.33	2.34	2.42	2.41	2.7	2.11*
**TG-HDL 4**	4.24	4.105	4.655	4.56	4.43	4.45	4.11	4.13	4.61	4.01*
**Chol-HDL 1**	13.02	13.39	11.66	12.2	12.16	11.22	15.5	12.56	13.99	14.35
**Chol-HDL 2**	7.025	7.035	6.32	6.5	6.06	6.01	6.89	6.59	6.97	6.63
**Chol-HDL 3**	9.975	10.31	9.855	9.015	10.11	10.07	9.29	9.04	9.67	10.20
**Chol-HDL 4**	21.77	21.55	23.45	22.535	21.37	21.18	18.94	18.97	22.83	21.55
**Free Chol-HDL 1**	3.39	3.24	3.01	3.025	3.9	3.2	3.65	3.37	3.74	3.79
**Free Chol-HDL 2**	1.73	1.68	1.445	1.435	1.76	1.58	1.58	1.49	1.8	1.69*
**Free Chol-HDL 3**	2.33	2.47	2.235	2.15	2.63	2.41	2.18	1.95	2.70	2.29*
**Free Chol-HDL 4**	4.57	4.475	4.42	4.125	4.42	4.12	3.99	3.63	4.97	4.41*
**Phospholipids-HDL 1**	14.3	15.81	13.175	12.78	14	12.5	16.92	14.62	15.17	15.3
**Phospholipids-HDL 2**	10.64	10.51	10.16	10.275	10.88	11.22	11.42	10.96	11.32	10.75
**Phospholipids-HDL 3**	15.59	15.15	15.72	14.98	16.84	17.06	15.06	14.85	15.94	16.25*
**Phospholipids-HDL 4**	29.9	29.39	30.865	30.44	29.54	29.34	27.36	26.74	31.34	29.25
**Apo A1-HDL 1**	16.82	16.82	15.95	15.48	13.65	13.99	17.88	16.69	13.33	15.95
**Apo A1-HDL 2**	16.43	17.87*	16.205	16.635	18.6	19.18	17.11	16	18.35	17.33
**Apo A1-HDL 3**	25.77	26.76	25.995	25.135	27.4	28.76	24.69	24.03	26.60	26.46*
**Apo A1-HDL 4**	81.00	80.94	86.72	85.84	84.15	81.3	75.14	74.75	83.71	82.01
**Apo A2-HDL 1**	1.71	1.97	1.66	1.695	1.81	2.01	2.10	2.13	2.04	2.065
**Apo A2-HDL 2**	2.98	3.19	2.955	3	3.56	3.61	3.76	3.5	3.74	3.57*
**Apo A2-HDL 3**	6.49	6.68	6.395	6.47	7.56	7.09	6.65	6.59	7.43	6.95*
**Apo A2-HDL 4**	22.02	22.15	23.18	23.08	22	21.77	19.59	20.16	23.46	23.05

Table S3. List of metabolites whose signals were assigned and integrated in the NMR spectra. DHA+OBG discrimination between T0 and T1 in all samples and only in milkshake; each significant comparison (T0 vs T1) is indicated with * for $p < 0.05$ and ** $p < 0.01$.

	**All BEF**		**Milkshake only**	
Metabolites	median T0	median T1	median T0	median T1
valine	0.2802	0.2971	0.2775	0.3329
isoleucine	0.0362	0.0395**	0.0405	0.0429
leucine	0.1123	0.1254*	0.112	0.1377
3-hydroxybutyrate	0.022	0.0208	0.0305	0.0222**
alanine	0.3994	0.4509*	0.3574	0.4676
acetate	0.0108	0.0147	0.0105	0.0136
glucose	0.8177	0.8947**	0.8368	0.968*
lactate	0.268	0.3168*	0.2501	0.3049
phenylalanine	0.0396	0.0474	0.04	0.0508
tyrosine	0.041	0.0482	0.0445	0.0545
creatine	0.0283	0.0361	0.0303	0.0344
creatinine	0.0613	0.063	0.0609	0.0598
glycine	0.1441	0.1693**	0.1407	0.1659**
glycerol	0.0159	0.0151	0.015	0.0136
histidine	0.0349	0.0348	0.038	0.037
formate	0.0025	0.0029*	0.0016	0.0026*
pyruvate	0.05	0.0558	0.0495	0.0554*
succinate	0.0141	0.0161	0.011	0.014
acetoacetate	0.0148	0.0286	0.0148	0.029*
dimethylamine	0.0086	0.012**	0.0084	0.013*
methionine	0.013	0.0207	0.0087	0.0308*
methanol	0.0524	0.0513	0.0478	0.0478
glutamine	0.0037	0.0068	0.0037	0.0077
lysine	0.0184	0.019	0.019	0.0192
proline	0.0135	0.0158	0.0115	0.0148
histidine	0.037	0.036	0.0376	0.0365

citrate	0.0216	0.0289**	0.0214	0.0328*
dimethylglycine	0.0128	0.0161	0.0114	0.0168
